# *In vivo* skin optical clearing efficacy quantification of clinically compatible agents using line-field confocal optical coherence tomography

**DOI:** 10.1117/1.JBO.28.5.055002

**Published:** 2023-05-27

**Authors:** Sergey M. Zaytsev, Marine Amouroux, Valery V. Tuchin, Elina A. Genina, Walter Blondel

**Affiliations:** a Université de Lorraine, CNRS, CRAN UMR 7039, Vandoeuvre-lès-Nancy, France; b Saratov State University, Science Medical Center and Institute of Physics, Saratov, Russia; c Tomsk State University, Laboratory of Laser Molecular Imaging and Machine Learning, Tomsk, Russia; d Institute of Precision Mechanics and Control, FRC “Saratov Scientific Centre of the Russian Academy of Sciences,” Saratov, Russia

**Keywords:** optical clearing, human skin, biocompatible optical clearing agent, line-field confocal optical coherence tomography, dermabrasion, sonophoresis

## Abstract

**Significance:**

The clinical use of optical methods for *in vivo* skin imaging is limited by skin strong scattering properties, which reduce image contrast and probing depth. The efficiency of optical methods can be improved by optical clearing (OC). However, for the use of OC agents (OCAs) in a clinical setting, compliance with acceptable non-toxic concentrations is required.

**Aim:**

OC of *in vivo* human skin, combined with physical and chemical methods to enhance skin permeability to OCAs, was performed to determine the clearing-effectiveness of biocompatible OCAs using line-field confocal optical coherence tomography (LC-OCT) imaging.

**Approach:**

Nine types of OCAs mixtures were used in association with dermabrasion and sonophoresis for OC protocol on three volunteers hand skin. From 3D images obtained every 5 min for 40 min, the intensity and contrast parameters were extracted to assess their changes during the clearing process and evaluate each OCAs mixture’s clearing efficacy.

**Results:**

The LC-OCT images average intensity and contrast increased over the entire skin depth with all OCAs. The best image contrast and intensity improvement was observed using the polyethylene glycol, oleic acid, and propylene glycol mixture.

**Conclusions:**

Complex OCAs featuring reduced component concentrations that meet drug regulation-established biocompatibility requirements were developed and proved to induce significant skin tissues clearing. By allowing deeper observations and higher contrast, such OCAs in combination with physical and chemical permeation enhancers may improve LC-OCT diagnostic efficacy.

## 1 Introduction

For the last few decades, optical spectro-imaging methods have been widely used in biology and medicine^1^. Among the wide range of applications, skin has become one of the main targeted structures, as their features such as non-invasiveness, high resolution, and speed of data acquisition made them a successful replacement or addition to traditional, often invasive and time-consuming methods, such as histological examinations. In particular, various spectroscopic methods, such as Raman, fluorescence, and diffuse reflectance spectroscopy, which allow quantitative and qualitative assessment of the biochemical structure of the studied tissues and their components, as well as imaging methods, such as confocal microscopy, multiphoton microscopy, second harmonic generation microscopy, and hyperspectral imaging, which allow for obtaining images of the studied tissues, are currently in clinical use for skin diagnostics and therapy.^1^^–^^3^

One of the widely used optical methods for skin imaging since the early 1990s is optical coherence tomography (OCT).^4^ This is an interferometric method in which the image of the tissue structure is obtained by registering low-coherent light backscattered or reflected by tissue.^1^^,^^5^ The original version of OCT was time-domain OCT (TD-OCT), which features fast translation of the internal interferometer’s reference mirror, allowing for the axial (i.e., perpendicular to skin surface) time-dependent scanning of skin.^1^ Full-field OCT was then developed, characterized by the ability to acquire “en face” (i.e., parallel to skin surface) skin images without the need to scan along the specimen.^6^ Another method is frequency- (or Fourier) domain OCT, featured by the absence of scanning of the reference mirror: scanning is carried out by the wavelengths of the source.^7^ Finally, a recently invented technique is line-field confocal OCT (LC-OCT) that combines the advantages of TD-OCT and confocal microscopy and provides 3D images of tissue at a sufficient resolution to distinguish the cellular structure of the skin.^8^

All techniques currently used in clinical environments for *in vivo* skin imaging at high spatial resolution do not properly meet the needs in dermatology for early non-invasive detection of all types of skin cancer (particularly melanoma) because of either insufficient resolution of the images (TD-OCT) or insufficient photon penetration depth in the tissue (in confocal and nonlinear microscopy). The lack of high-resolution vertically oriented cross-sectional views is also a limitation in confocal and nonlinear microscopy.

Also, the use of OCT for skin imaging, as well as other optical techniques, is limited by strong light scattering by biological tissues.^9^ Thus, the resolution, contrast, and consequently, the diagnostic potential of this method are greatly reduced. The main reason for the strong scattering of biological tissues is the difference in the refractive indices (RI) of the tissue components and the interstitial fluid.^1^ Now, a widely investigated method to increase the refractive index of the interstitial fluid and thus reduce scattering is tissue optical clearing (OC).^10^^,^^11^ It is based on the use of chemical agents whose refractive indices are close to those of the structural components of biological tissue. Due to their immersion as a result of surface application or injection into biological tissue, they cause partial dehydration of the skin, and then replace the interstitial fluid. As a result of the action of these substances, called OC agents (OCAs), the scattering of biological tissues is reduced, which leads to an increase in the resolution and contrast of optical methods^10^^,^^12^^,^^13^.

At the moment, there are many examples of using an OC technique to improve the contrast of OCT skin images *in vivo*^10^^,^^11^^,^^14^^–^^16^ and, in particular, its application for skin cancer characterization.^17^ However, when translating these methods into clinical use, there is a need to comply with established regulations, including the use of chemicals on healthy and pathologically changed skin of patients. At high concentrations, OCA show significant results; however, in clinical settings, the concentrations of the substances used may need to be reduced to pass the threshold of clinical utility and biocompatibility.^18^

The penetration of most chemicals is hindered, as they are stopped by the stratum corneum (SC) layer, which acts as a natural barrier of human body.^19^^–^^21^ Most of the used OCAs are hydrophilic and are used in high concentrations, which hinders their penetration through the SC into the living epidermis,^22^ but at the same time they may cause damage and even necrosis of the skin when injected under the dermis.^23^^,^^24^ However, a low concentration of OCA does not allow it to achieve a noticeable clearing effect. Thus, the use of OCA at biocompatible concentrations should be combined with the use of substances called chemical permeation enhancers (CPEs), which, even at relatively low allowed concentrations, can disrupt the impermeable structure of the SC and facilitate the penetration of OCA. In the literature, alcohols^25^ and organic solvents, such as dimethyl sulfoxide (DMSO),^26^^,^^27^ thiazone,^28^ and fatty acids (linoleic and oleic)^26^^,^^29^ are most often used as CPE.

Additionally, a large number of physical methods for enhancing the skin permeability have been described in the literature.^30^^–^^37^ Among them, microdermabrasion^31^ and sonophoresis^36^^,^^37^ can be distinguished as minimally invasive. They can be combined with CPEs for more efficient biocompatible clearing.

The aim of the current study was to experimentally evaluate the efficacy of optical clearing of human skin tissue *in vivo* using biocompatible OCA combined with chemical and physical permeability enhancers based on LC-OCT imaging.

## 2 Materials and Methods

### 2.1 Chemical Agents

In this study, nine different combinations of OCA and CPEs were studied as potential clinically biocompatible mixtures. The latter were chosen based on the literature data including our own preliminary studies.^1^^,^^10^^,^^13^^,^^38^ Three chemicals, most often referred to as OCA in the scientific literature, were selected: polyethylene glycol 400 (PEG, Sigma-Aldrich, United States) as an OCA from the group of alcohols and two aqueous 3M-solutions of sugars—sucrose (Sigma-Aldrich, United States) and glucose (Sigma-Aldrich, United States). Three other chemicals, mentioned as CPE in the scientific literature,^1^^,^^13^ were considered: propylene glycol (PG, Sigma-Aldrich, United States) as an alcohol group representative (DMSO, Sigma-Aldrich, United States) as an organic solvent and Oleic acid (OA, Sigma-Aldrich, United States) as the fatty acid. For the sake of biocompatibility, the concentrations of each of the aforementioned substances in the present experimental study did not exceed their maximum allowed concentration, established by the Food and Drug Administration (FDA) and contained in the inactive ingredients database, for topical application in the form of a solution.^18^ The resulting compositions of OCA and CPE, as well as their volume fractions and maximum allowed concentrations are shown in Table 1. As there is no information about maximum FDA-allowed concentration for topical application of glucose and sucrose, a concentration value (v/v) of 50% was established for our experiments as it was previously reported as the most effective concentration of glucose for OC.^39^ In the case of oleic acid and DMSO, it was not possible to mix each of them (alone) with any of the three OCA since in this case the maximum allowed concentration for these chemicals would be exceeded. For this purpose, PG or distilled water was additionally mixed as the second CPE.

**Table 1:** Concentration values of the nine mixtures of OCA and CPEs with corresponding FDA-allowed maximal concentrations for topical application in the form of solution and volume fractions (%, v/v) of resulting mixtures.

Concentration (%, v/v)		OCA			CPE			Distilled water
		Glucose	PEG-400	Sucrose	DMSO	OA	PG	
	Max *c*. (FDA)	—	4 (w/v)	—	45,5 (w/w)	7,4 (w/w)	99,98 (w/v)	—
Glucose/DMSO		50			45,5			4,5
Glucose/OA/PG		50				7,44	42,56	
Glucose/PG		50					50	
PEG/OA/PG			3,52			7,44	89,04	
PEG/PG			3,52				92,48	
PEG/PG/DMSO			3,52		45,5		50,98	
Sucrose/DMSO				50	45,5			4,5
Sucrose/OA/PG				50		7,44	42,56	
Sucrose/PG				50			50	

### 2.2 Anatomical Skin Sites

The human skin sites under investigation were the areas of left- and right-hand dorsum skin *in vivo* between the thumb and forefinger of healthy volunteers. Three volunteers aged between 25 and 31 years with the skin phototype of 2 and 3 were enrolled in this study. All skin areas were subjected to the experimental protocol repeatedly due to the number of mixtures and experimental conditions. To prevent residual effects of the previous protocol, each skin area remained intact for a week before the beginning of the next exposure. All volunteers gave their informed consent for topical application of OCAs mixtures, dermabrasion, and sonophoresis of skin dorsum of their hand and the acquisition of LC-OCT images, for the kinetics study. Volunteers’ safety was guaranteed thanks to FDA-approved concentrations of OCAs and to CE-marked LC-OCT, dermabrasion, and sonophoresis medical devices. An authorization for the human skin studies *in vivo* was obtained from the Saratov State Medical University Ethical Committee (protocol no. 11 by 7 June 2022).

### 2.3 Technical Equipment

#### 2.3.1 LC-OCT

**Fig. 1:**
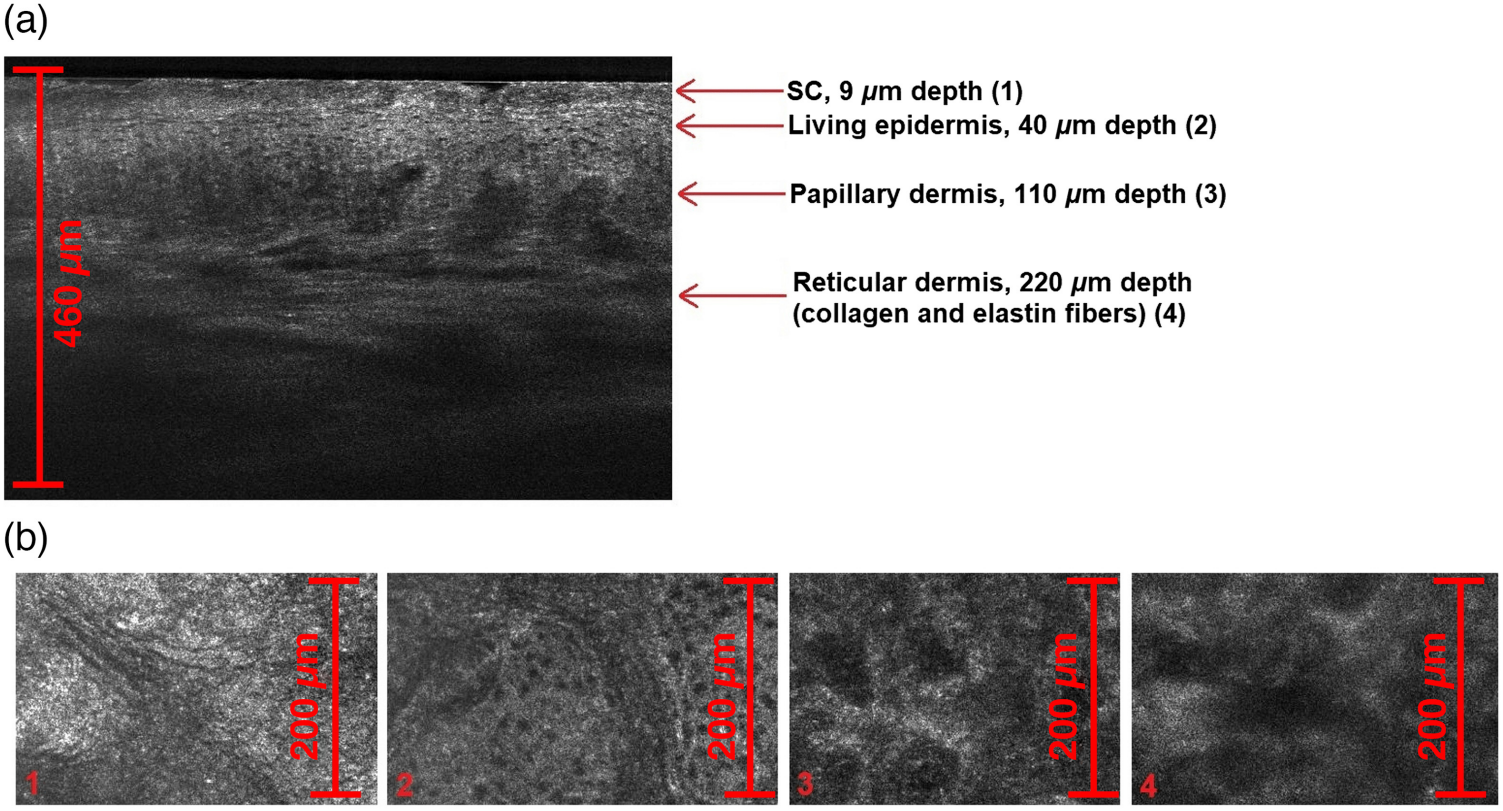
(a) Histology-like B-scan extracted from the LC-OCT acquired 3D image of human intact skin *in vivo*, displaying visible distinguishing between different skin layers. (b) Four different skin layers images, extracted as confocal-like horizontal scans from the 3D image: SC layer, acquired at 9-μm depth (1), living epidermis, acquired at 40-μm depth (2), papillary dermis, acquired at 110-μm depth (3), and reticular dermis, acquired at 220-μm depth (4).

The LC-OCT device “deepLive” (Damae Medical, France) was used for the image acquisition. Operating at a 650 to 950 nm spectral range emitted by a supercontinuum laser, it provides a unique 3D imaging modality, allowing one to switch from a histology-like vertical mode to a confocal-like horizontal mode and to record a 3D stack of tissue volumes *in situ* with a maximum axial and lateral resolution of <1.3  μm and a penetration depth about 500  μm.^8^ Such a resolution allows estimating the contrast changes of the tested skin caused by OC at a cellular level (Fig. 1).

#### 2.3.2 Physical permeation enhancers

Therapeutic ultrasound (US) (Pulson 100, Gymna, Belgium) was used as a physical permeability enhancer in experiments with OCT since this technique helps to increase the permeability of the skin.^36^^,^^37^^,^^40^ The duty cycle was 100%, frequency was 1 MHz, and the power density was $1\;W/{\mathrm{cm}}^{2}$. The second physical permeability enhancer was microdermabrasion (Philips VisaCare, Philips, Netherlands). This procedure is widely used in cosmetology and involves the abrasion/removing of the SC but does not damage the living epidermis that leads to increased penetration of OCA into the skin. It was recently shown in Ref. 37 that 1.5-min dermabrasion removed only 8±4  μm of the SC; at the same time, erythema, as well as discomfort, were absent.

### 2.4 Experimental Protocol

**Fig. 2:**
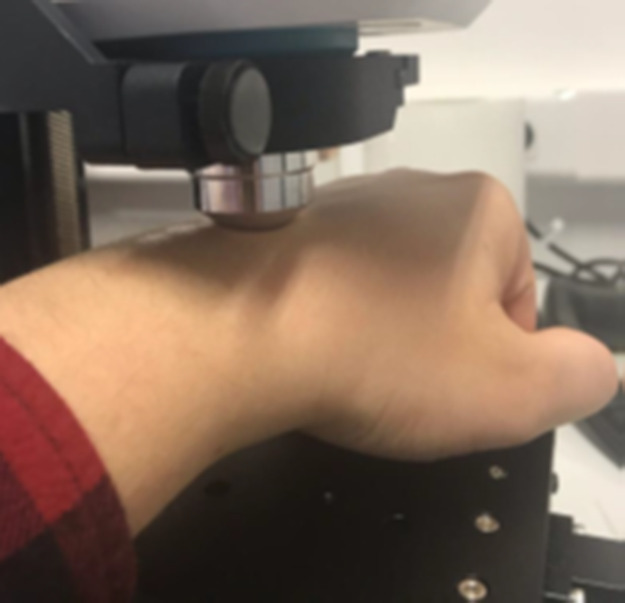
Photograph of the tested skin site in gentle contact with LC-OCT probe.

The skin sites were prior cleaned with ethanol to remove fat residuals. A starting LC-OCT 3D image of the latter cleaned intact skin was first taken (t=0 time point) (Fig. 2). Then, the investigated area was treated for 1 min using a dermabrasion device followed by another 3D image acquisition. Afterwards, at time point t=1  min, OCA was topically applied on the selected skin site, which was then exposed to therapeutic US for two consecutive sequences of 5 min duration each. Between and after the US treatments intervals (at t=6 and 11 min, respectively), and then every 5 min for 30 min (t=16, 21, 26, 31, 36 and 41 min), 3D images of the selected area were taken using LC-OCT. Note that in those conditions the OCA mixture served as the immersion liquid for the LC-OCT probe during every successive image acquisition (including intact and after dermabrasion). Control measurements were performed at identical time points (t=0 to 41 min) on a similar skin site only subjected to paraffin oil as the LC-OCT probe immersion liquid. The skin site in control measurements was not exposed to any of the external influences (neither dermabrasion nor sonophoresis) described in the protocol for OCA.

### 2.5 Data Analysis

From the obtained 3D images, the entire volume of in-depth data was taken for analysis, except for the superficial 36-μm-thick layer. This was done to ignore the superficial areas of high contrast caused by reflection from the glass interface of the LC-OCT probe, which is configured with a slight slope. Thus, the 20-μm-thick SC layer^41^ was mostly not included in the analysis, and the surface of the resulting image was represented by the stratum granulosum.

**Fig. 3:**
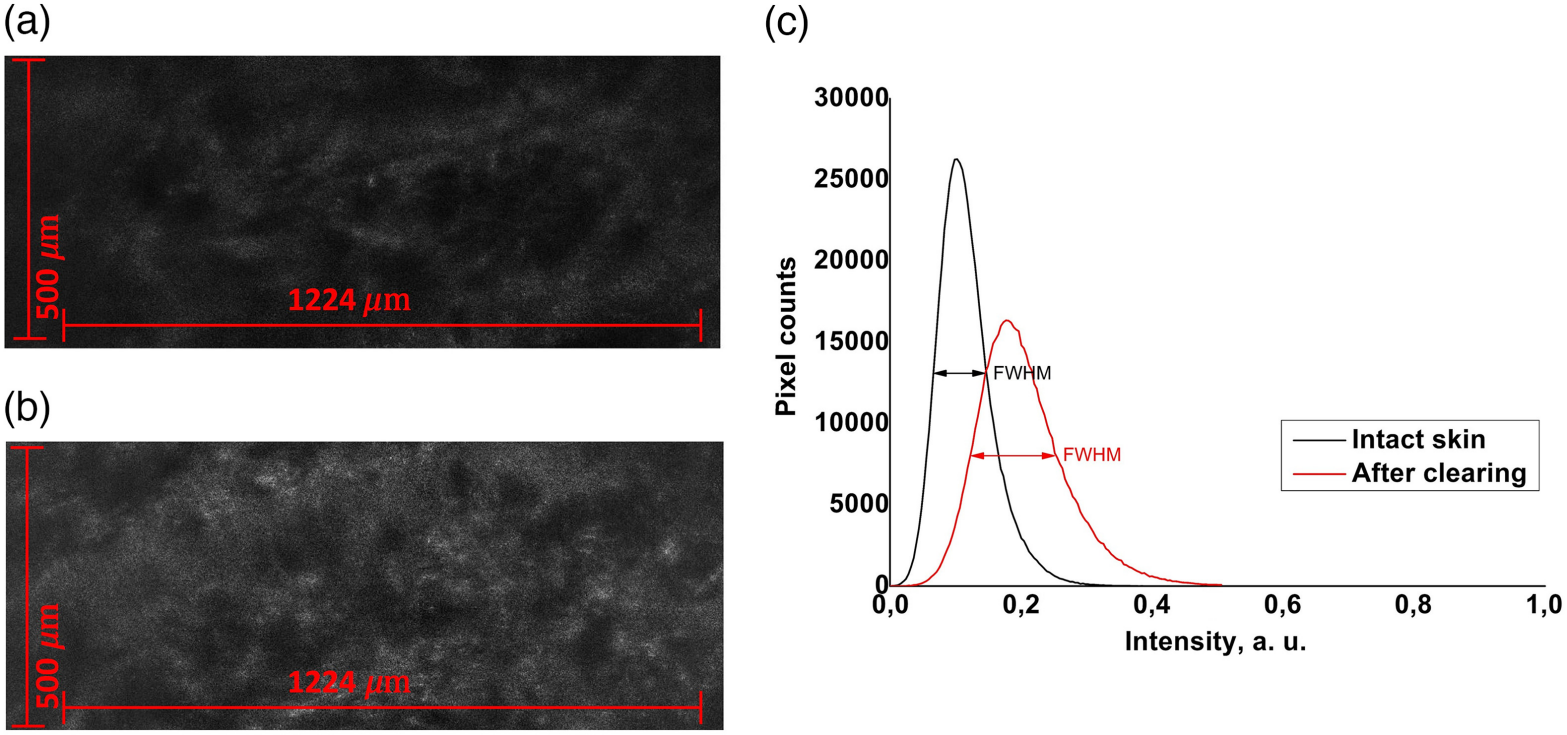
Examples of individual horizontal 1224×500  μm sections extracted from LC-OCT 3D images at 200-μm depth from intact skin (a) and from skin after OC (b); (c) corresponding graphs of the pixel intensity distribution in images at a 200-μm depth for intact skin (black) and for skin after OC (red). Corresponding arrows show the FWHM for each curve.

For data analysis, the pixel intensity distribution was calculated for each horizontal section (1224×500  pixels) of the 1224×500×460  μm 3D image (Fig. 3) along the z-axis with a depth step of 10  μm.

**Fig. 4:**
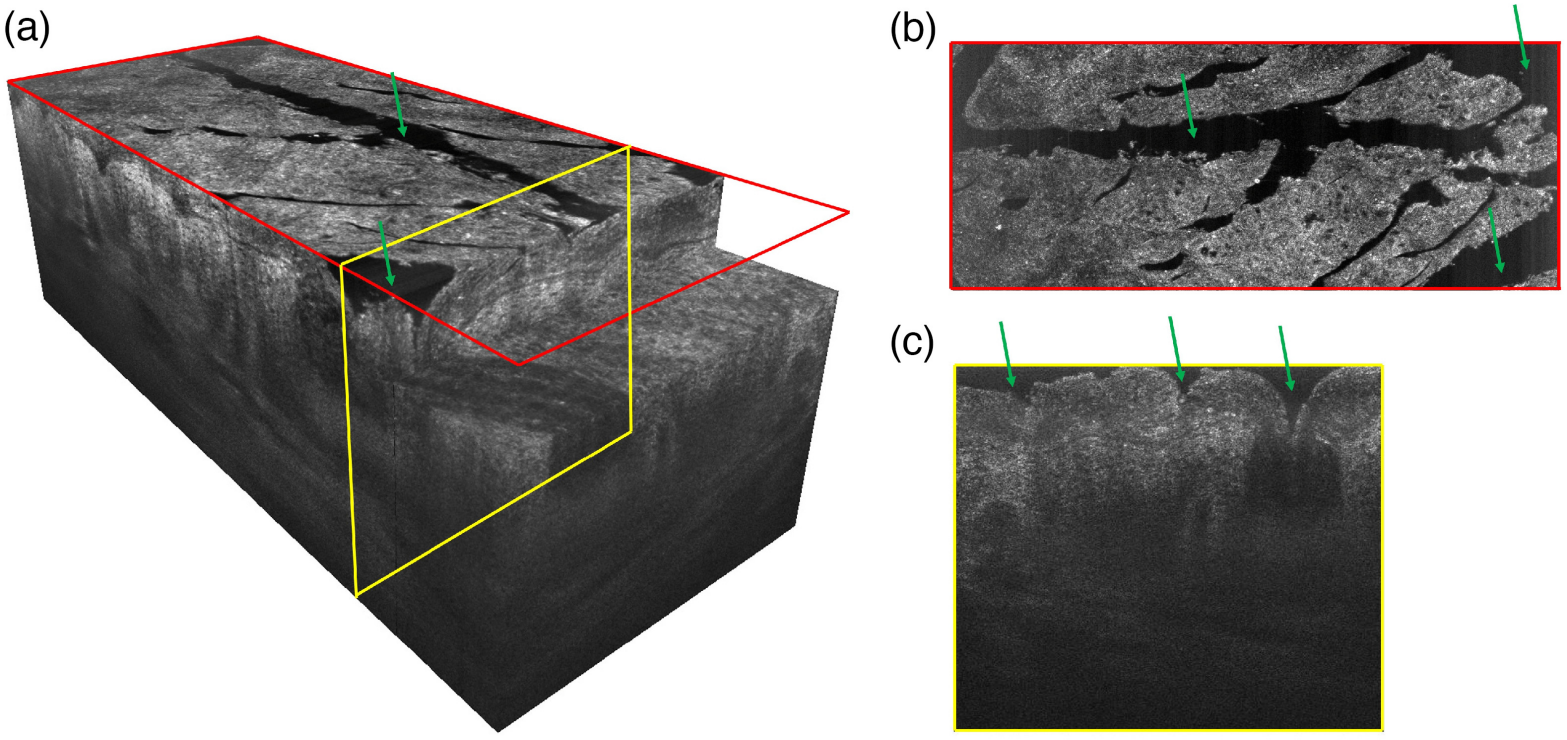
Illustration of a typical 1224×500×460  μm 3D image of skin (a), acquired by LC-OCT (deepLive, Damae Medical, France) in the current study and corresponding “slices” of a surface (b) and in-depth sections (c) showing the presence of the skin relief-related hollow dark regions (green arrows).

Note that the pixel intensity histograms obtained for most of the individual horizontal sections of 3D images had the form of a normal distribution, except for the uppermost skin layer images where secondary peaks appeared due to hollow black areas on images related to the skin relief (Fig. 4). The peak intensity and the full width at half maximum (FWHM) values of such a distribution make it possible to estimate, respectively, the average intensity and image contrast of an individual horizontal section. To simultaneously assess the effect of OC on the overall intensity and contrast of the horizontal section, the ratio R between mean intensity $I_{\text{mean}}$ and FWHM was calculated as

$$R=\frac{I_{\text{mean}}}{1-\mathrm{FWHM}}.$$ (1)

**Fig. 5:**
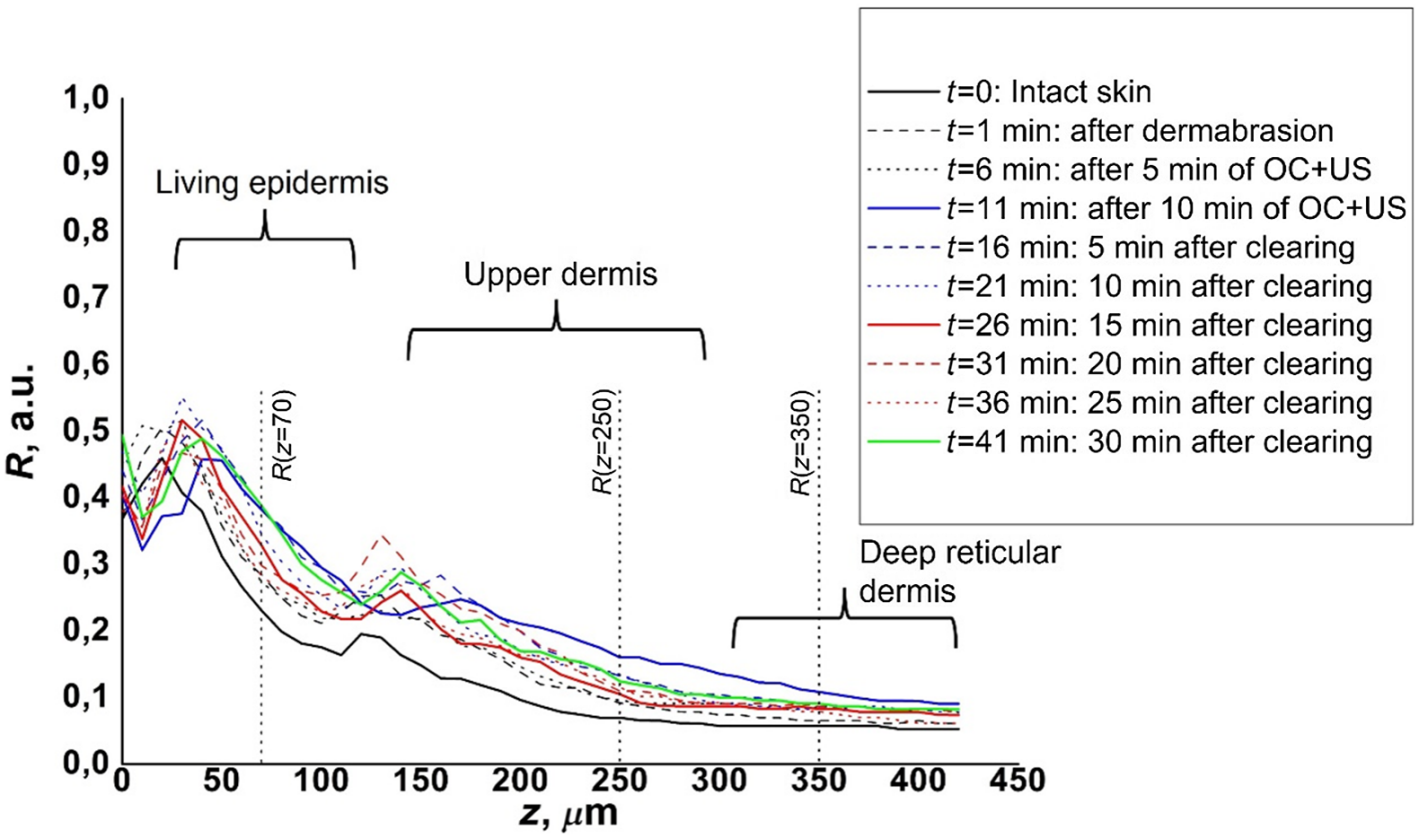
Illustration of depth dynamic of ratio R for different experimental protocol steps for one OCA, measured on one volunteer. The three vertical dotted black lines indicate the depths within the three different skin layers (shown with curly brackets) at which the changes of R were calculated.

To estimate the ability and rate of OCA penetration into the superficial and deep layers of the skin, the relative changes in percentages of R compared to the initial values (at t=0) were calculated for measurement steps t=6, 11, and 26 min (Fig. 5), where t=6 is LC-OCT measurement after 5 min of US-assisted OC (OC+US), t=11—after 10 minutes of OC+US, t=26−15  min after OC. These ratios were calculated at 70, 250, and 350  μm depths, in the midst of the living epidermis, the upper dermis, and the deep reticular dermis layers, respectively. Then, these ratios were averaged among the volunteers.

Then, the area under curve (AUC) was calculated for the depth dependent graphs of *in vivo* skin R ratio in the range 70 to 400  μm. This value allows to evaluate the overall increase in contrast and intensity of the 3D image.^1^ The range between 0 and 70  μm was excluded from AUC calculation because in the upper skin areas artifacts in the R-values were observed, caused by the presence of the hollow areas in the horizontal sections described above. The obtained AUC values were averaged between the volunteers with respect to time points and OCA mixture. To compare the effectiveness of the different OCAs to each other as well as with control results, average AUC values previously calculated were all normalized to their corresponding initial values (at t=0), i.e., AUC relative values. Image processing and calculations were performed on MATLAB (R2018a, The Mathworks).

### 2.6 Exponential Fitting of AUC Data

The experimental protocol conducted in this study can be nominally divided in time into two phases. The first phase corresponds to the “active part” of the protocol in which all external manipulations (dermabrasion, US treatment) and, consequently, a major OC contribution were performed, and takes a time range t from 0 to 11 min. The rest of the protocol involved only “passive” observation and measurements for 30 min (from t=11  min to t=41  min), and thus the active manipulations here have already been completed and any changes in AUC can be mainly associated only with passive diffusion of OCA into the skin. To estimate the overall completion of the OC process after the end of the active phase, which would be an undoubted advantage of a specific OCA since in this case additional “passive” phase is not required, the experimental data were fitted. Taking into account the nominal separation of the experimental protocol into two phases described above, the biphasic exponential association was chosen as an appropriate model. It is described by the function

$$y=\{\begin{array}{ll} Y_{b} & x<{\mathrm{TD}}_{1}\\ Y_{b}+A_{1}(1-e^{-\frac{x-{\mathrm{TD}}_{1}}{Tau_{1}}}) & {\mathrm{TD}}_{1}\leq x<{\mathrm{TD}}_{2}\\ Y_{b}+A_{1}(1-e^{-\frac{x-{\mathrm{TD}}_{1}}{Tau_{1}}})+A_{2}(1-e^{-\frac{x-{\mathrm{TD}}_{2}}{Tau_{2}}}) & x\geq {\mathrm{TD}}_{2} \end{array},$$ (2)

where $Y_{b}$ is the y value at which exponential begins; $A_{1}$ and $A_{2}$ are the first and the second exponential amplitudes, respectively; ${\mathrm{TD}}_{1}$ and ${\mathrm{TD}}_{2}$ are the first and the second time offset, respectively; and $Tau_{1}$ and $Tau_{2}$ are the first exponential and the second exponential time constants, respectively.

Since we assumed the hypothesis that the “passive” phase of our experimental protocol is a logical continuation of the “active,” and therefore, its amplitude should remain unchanged or increase (an insufficient time will pass for a significant reversal of the OC effect due to the *in vivo* physiological response), we determined for this model the lower bound of the $A_{2}$ parameter not lower than zero ($A_{2}\geq 0$). $Y_{b}$ parameter was fixed as 1 for our model, as the fitted AUC data were normalized to initial value for all OCA. ${\mathrm{TD}}_{1}$ and ${\mathrm{TD}}_{2}$ parameters were manually fixed as 0 and 11 min, respectively, as the “active” phase starts at t=0 timepoint and the “passive” phase of our hypothesized biphasic model starts right after all the manipulations are done (t=11  min). The fitting was performed in Origin 2018 (OriginLab Corporation).

To compare simultaneously the amplitude and the time constant of the fitted kinetic AUC data, two ratios were calculated for each dataset. The first one is the slope at origin fitted curve for the first phase (${\mathrm{SAO}}_{1}$), defined as ${\mathrm{SAO}}_{1}=A_{1}/Tau_{1}$. For the second “passive” exponential, an ${\mathrm{SAO}}_{2}$ ratio was defined as ${\mathrm{SAO}}_{2}=A_{2}/Tau_{2}$.

## 3 Results and Discussion

**Fig. 6:**
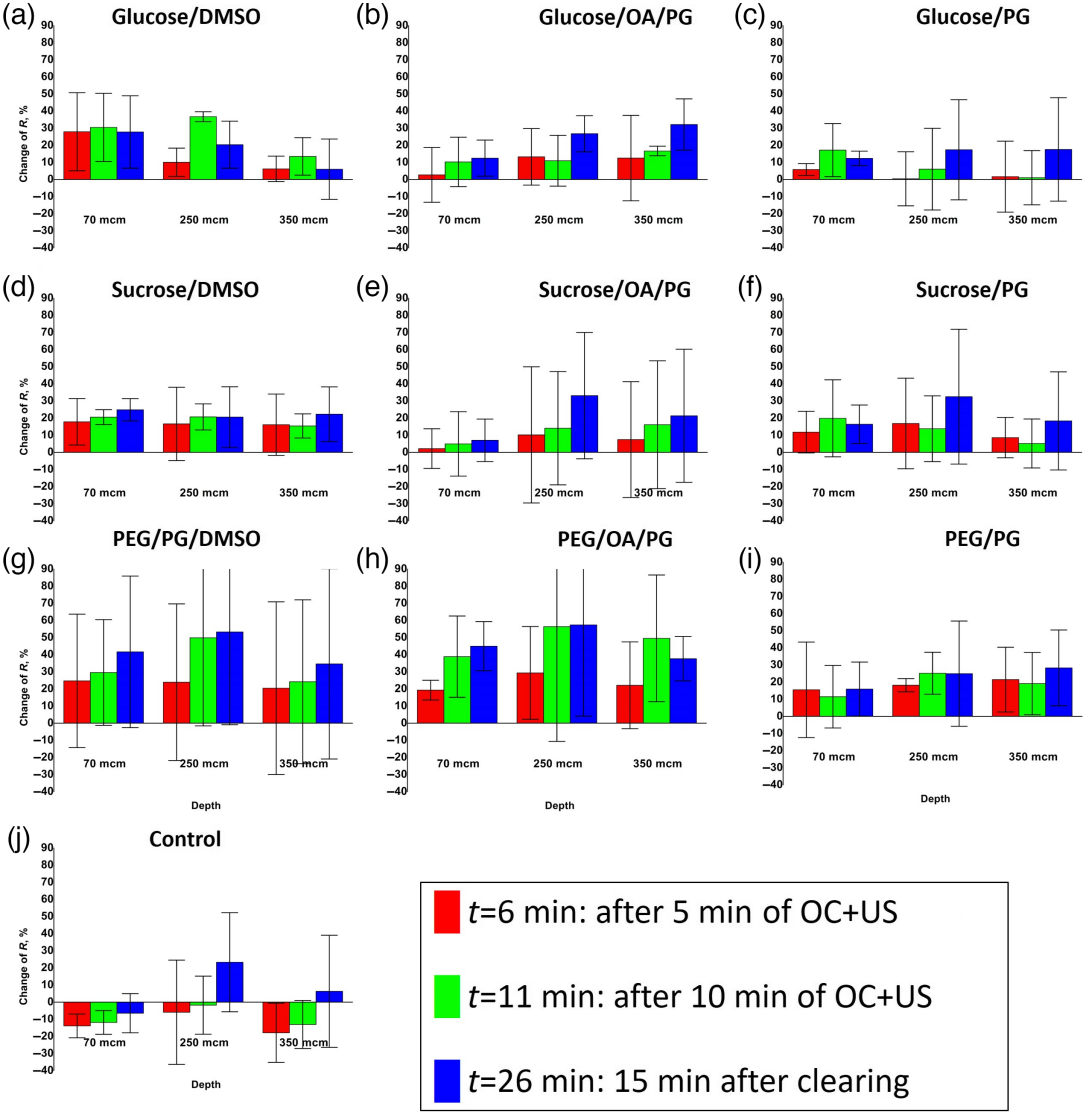
Percentages of R relative changes (with reference to initial value at t=0): after 5 min of OC and US (red) and after 10 min of OC and US (green) and 15 min after OC (blue). Average ± SD values are given among the volunteers for the 10 different OC mixtures: (a) glucose/DMSO, (b) glucose/OA/PG, (c) glucose/PG, (d) sucrose/DMSO, (e) sucrose/OA/PG, (f) sucrose/PG, (g) PEG/PG/DMSO, (h) PEG/OA/PG, (i) PEG/PG, and and (j) control condition.

The average relative changes of R values at three different skin depths for each experimental condition (each OCA) and for the control condition are presented in Fig. 6. The SD bars represent the standard deviation due to the biological variation of *in vivo* skin samples and also due to the fact that individual horizontal sections were used without averaging over the skin volume.

From the plots shown in Fig. 6, it can be observed that the control experiment [Fig. 6(j)], as expected, did not cause any significant increase in R whatever the analyzed depth: mean change of R at t=11  min is −9%. The use of DMSO [Figs. 6(a), 6(d) and 6(g)] as a CPE resulted in an increase in R at 70-μm depth (mean 27% increase at t=11  min). For mixtures of DMSO with both sugars, there was a rise in R after the first 5 min of US-assisted OC (28% and 18%—R increase for mixtures of glucose/DMSO and sucrose/DMSO, respectively), and then no significant increase in subsequent time periods (for glucose/DMSO, the ratio R did not change 15 min after OC, for sucrose/DMSO we observed a 7% increase). Nevertheless, the use of PEG with DMSO in combination with PG as an additional CPE resulted in a 25% R increase after 5 min of clearing, and at 15 min after clearing it was still possible to observe a 17% R increase (up to a total 42% increase). A similar trend was observed in the deeper layers of the skin. At 250- and 350-μm depths, the PEG/PG/DMSO mixture resulted in 24% and 20% R increase, respectively, after 5 min of US clearing. Then, 15 min after clearing, the R ratio rose again to 53% and 35%, respectively. The sucrose/DMSO mixture here also caused a clearing effect, expressed as an increase of the R parameter, but on a smaller scale and without any increase with time. Remarkably, the glucose/DMSO mixture showed a very slight R increase at a 350-μm depth; however, at a 250-μm depth, the R ratio was modified (27% increase) after the second 5-min US clearing compared to the first one. This allows one to conclude that the dynamics of glucose/DMSO penetration into the deep layers of *in vivo* skin is lower compared to the PEG/PG/DMSO mixture.

This is an interesting observation since the volume concentration of both sugars in OCA was several times higher than that of PEG. DMSO as CPE can interact with the lipid layers of the SC and thus facilitate the penetration of hydrophilic OCAs, such as sugars and PEG.^1^^,^^13^^,^^42^ It is likely that in our case the effect of DMSO on the increase in permeability *in vivo* was limited, which did not lead to a strong subsequent long-term increase in OC. The PG used in the PEG/PG/DMSO mixture is also able to interact with and dissolve SC lipids, accelerating skin dehydration under the action of hyperosmotic agents and increasing the rate of OC.^10^^,^^43^ Probably, the combination of these two CPEs made it possible to obtain an enhanced in-depth clearing effect that continued with time, even when using lower concentrations of the hyperosmotic agent. Moreover, PG and DMSO cannot be strictly classified as CPE since they also possess the properties of OCA.^10^^,^^13^

With the use of PG as CPE, the clearing effect for glucose and sucrose mixtures was mainly limited by the upper skin layers and did not increase much with time (at a 70-μm depth, the change in R, 15 min after OC, compared to the first 5 min of US clearing was 6% and 4% for mixtures of glucose/PG and sucrose/PG, respectively). Compared to sugar mixtures, the PEG/PG mixture showed a marked clearing effect even in the deep reticular dermis. At a 350-μm depth, the R ratio increased by 21% after 5 min of US-assisted clearing, and then, after 15 min, it continued to slightly increase to 28%. Compared to the PEG/PG/DMSO mixture results described above, the PEG/PG mixture showed clearing potential in the deep layers of *in vivo* skin, albeit on a smaller scale. This is probably due to the lack of penetration enhancing properties of DMSO,^42^ although PG was used in this case at a high (92% vol./vol,) concentration.

Mixtures of sugars with a paired OA/PG used as CPE showed a better clearing effect in depth than with the other enhancers (mixed with DMSO). Even though the change in R in the epidermal layer at a 70-μm depth was not as significant as the one obtained with the other sugar/enhancer mixtures, at a 250-μm depth, the mixtures of Glucose/OA/PG and Sucrose/OA/PG showed a steady increase during the experiment. For the Glucose/OA/PG mixture, this ratio continued to increase by 15% 15 min after the end of clearing compared to this value 5 min after US clearing. The similar increase for the sucrose/OA/PG mixture was observed with a value of 22%. Moreover, this trend was also found in the deep layers of the dermis. At 350-μm depth, the relative change in the R ratio for the Glucose/OA/PG mixture increased by a factor of almost 2.5 from 13% after 5 min of OC and US to 32% 15 min after the end of US-assisted clearing that is 25 min after OC start. The same values for a mixture of sucrose/OA/PG showed a three-fold increase from 7% to 21%. This difference from the results obtained without the use of oleic acid as CPE indicates the advantage of using it with the sugars to clear the “deep” layers of *in vivo* skin. OA, as well as DMSO and PG, can increase the permeability of the SC for OCA by disrupting the organization of its lipid layer.^29^^,^^44^^,^^45^ However, OA does not have hyperosmotic properties, and therefore does not cause much dehydration of the skin, unlike other CPEs. This fact, as well as, probably, a larger permeability increase of the SC layer leads to a deeper penetration of the OCA into the skin and, as a result, a better increase in the contrast and intensity of images throughout the depth.

Based on the results presented in Fig. 6, the best OC effect across the depth of *in vivo* skin was achieved using a mixture of PEG/OA/PG. In the epidermal layer at a 70-μm depth, the relative change between R 15 min after OC and R after 5 min of OC + US clearing was 26% (from 19% to 45%). This is the highest value of R change achieved at this depth under the observed parameters. At the same time, in the dermis layer at depths of 250 and 350  μm, the change in R reached its maximum value already after 10-minutes of US-assisted clearing and amounted to 56% and 50%, respectively.

**Fig. 7:**
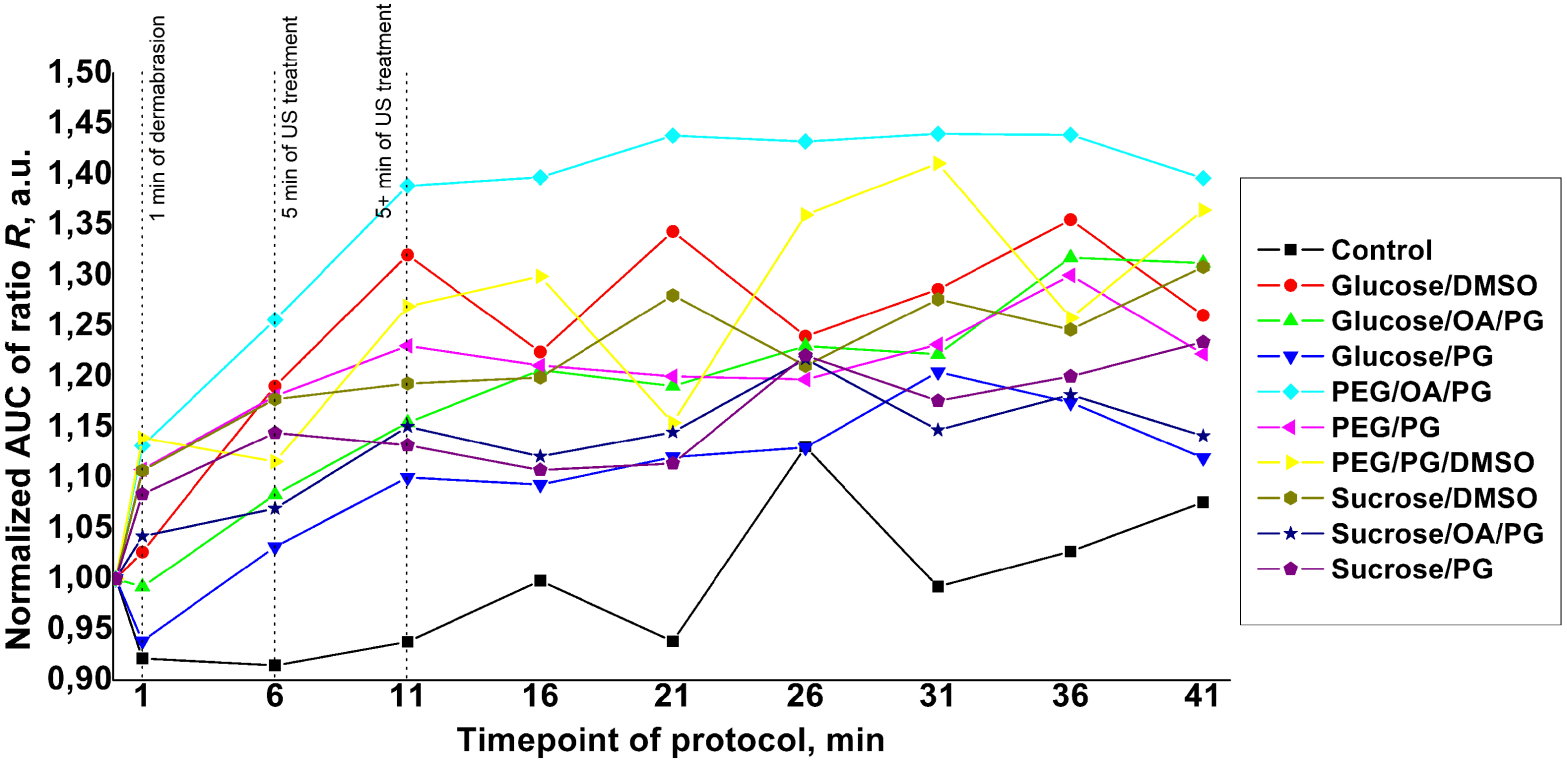
Normalized mean AUC (n=3) variation kinetics of the R ratio for the nine different OCA mixtures and for the control condition.

Averaged (n=3 volunteers) AUC kinetic curves of ratio R (70 to 400  μm) and normalized to the corresponding initial values for each OC and control protocol are presented in Fig. 7. The SD bars were removed to keep the curves legible: the mean SD value is 10% of non-normalized initial values.

These results confirm the observation and analysis conducted from Fig. 6. The PEG/OA/PG mixture showed the most confident kinetics and reached an AUC value of ∼1.4 (corresponding to a 40% increase) already at the end of the 10-min clearing+US treatment. Then the changes were insignificant, which indicates the completion of the clearing process. The glucose/DMSO mixture showed a similar value at the same time point (∼1.32), but as shown in Fig. 6, the clearing effect in the deeper skin layers was more pronounced when PEG/OA/PG was used as the OCA. These results are in good agreement with results referenced in Ref. 37, where the combined use of microdermabrasion, US-treatment, and oleic acid as an OCA on *in vivo* human skin resulted in more than a twofold increase in OCT probing depth and more than a threefold increase in OCT signal amplitude.

**Fig. 8:**
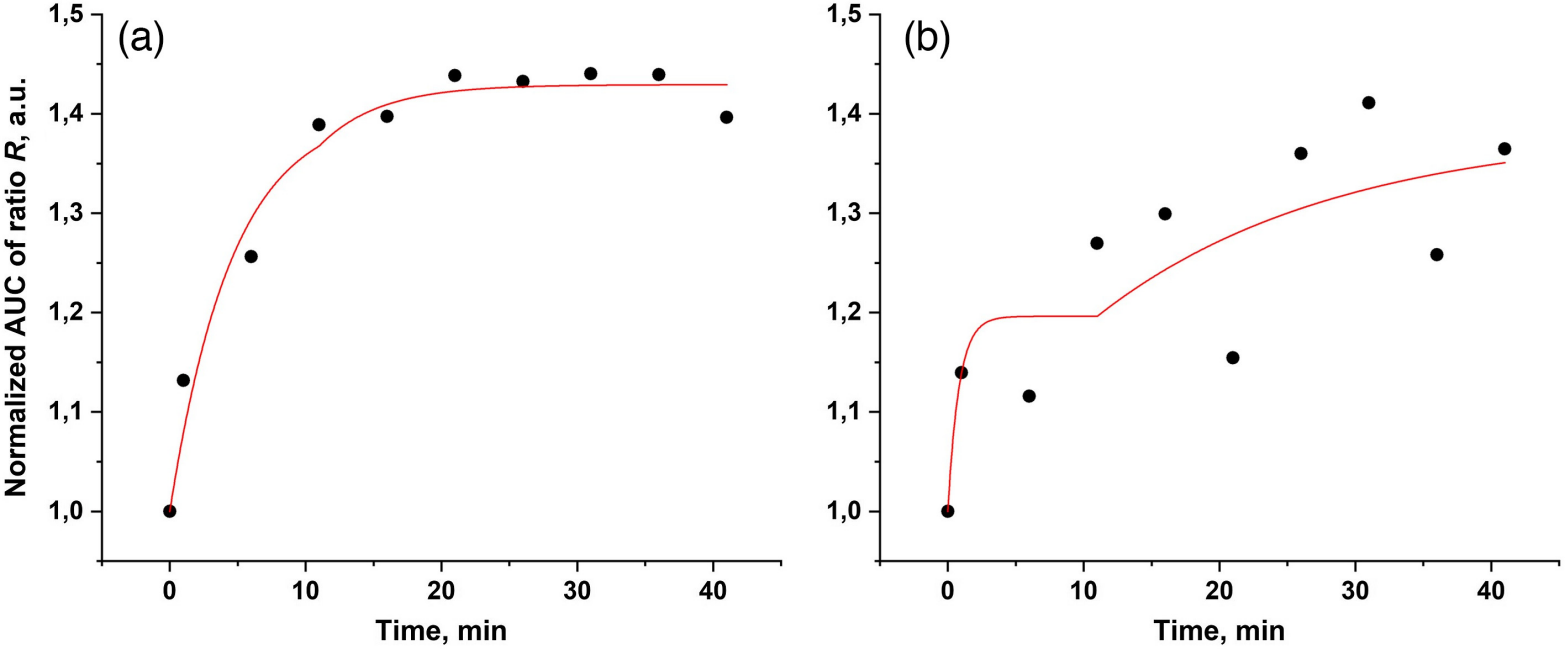
Experimental data (black circles) of the AUC of ratio R time dependence and the corresponding fitted curves (red line) of the biphasic exponential model [Eq. (2)] for the mixture of (a) PEG/OA/PG, which showed the highest $r^{2}$ value (0.97) and for the mixture of (b) PEG/PG/DMSO, which showed the lowest $r^{2}$ value (0.69). In this model, the lower bound of the $A_{2}$ parameter was fixed to be not lower than zero ($A_{2}\geq 0$). $Y_{b}$ parameter was fixed as 1, and ${\mathrm{TD}}_{1}$ and ${\mathrm{TD}}_{2}$ parameters were manually fixed as 0 and 11 min, respectively.

Kinetics of AUC of ratio R fitted with a biphasic exponential model showed high values of the coefficient of determination $r^{2}$. Depending on the OCA used, $r^{2}$ took values in the range from 0.69 to 0.97 (Fig. 8).

**Fig. 9:**
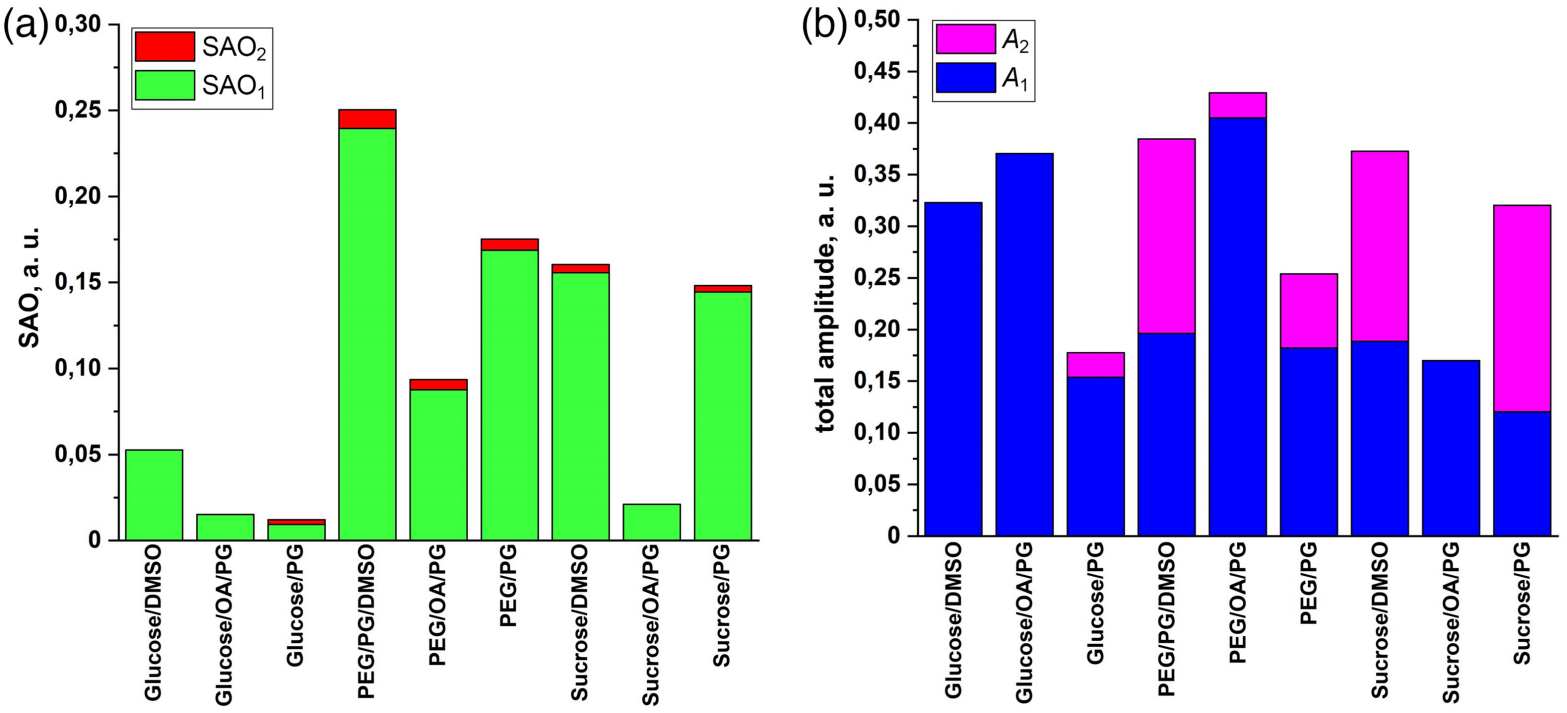
Stacked histograms of (a) calculated ${\mathrm{SAO}}_{1}$ and ${\mathrm{SAO}}_{2}$ parameters and (b) derived $A_{1}$ and $A_{2}$ parameters of fitted curves of biphasic exponential association model.

The results of the biphasic exponential model-derived parameter analysis are presented in Fig. 9.

These results largely agree with the results presented in Figs. 6 and 7. The PEG/PG/DMSO mixture shows the highest stacked value of the SAO parameter; moreover, the main contribution to the column is made by the ${\mathrm{SAO}}_{1}$, which indicates the greatest efficiency of this OCA during the “active” phase of the biphasic exponential model in terms of the balance between achieved amplitude and the time spent on this. Quite similar results were achieved using the PEG/PG, sucrose/DMSO, and sucrose/PG mixtures. However, when looking at the stacked histograms of the exponential curves total amplitude, it becomes noticeable that for the above OCAs, a significant contribution to the amplitude is made by the parameter $A_{2}$, which corresponds to the amplitude of the “passive” phase. Thus, their relatively small ${\mathrm{SAO}}_{2}$ values and corresponding significant $A_{2}$ values indicate a long “passive” phase before the OC reaches its final plateau, which is not the optimal expected result.

On the contrary, mixtures of glucose/DMSO, glucose/OA/PG, and PEG/OA/PG showed the highest values of the total amplitude, and the main contribution to the amplitude was made by parameter $A_{1}$, which indicates their effectiveness in terms of the duration of the process—OC was for the most part completed within 11 min of the “active” phase, when all the manipulations described in the experimental protocol were completed. Comparison of the actual results of the AUC of ratio R kinetics and the amplitudes of the exponential curve of the hypothesized biphasic model clearly shows that the PEG/OA/PG mixture has the best values of the increase in contrast and intensity of the signal obtained by LC-OCT in terms of the process time.

Thus, we have experimentally shown the potential of OC of human skin *in vivo*, enhanced by chemical and physical methods of permeability enhancement, by biocompatible OCA, which makes it possible to safely use this technique for the diagnosis and treatment of healthy and pathologically altered skin areas.

Further prospects for research in this direction should imply optimization of the biocompatible OCA and CPEs mixture composition by expanding the list of chemicals under study, as well as the inclusion of this technique in real clinical studies of neoplastic skin lesions. To improve the diagnostic ability of the optical method of skin analysis, it also makes sense to use a multimodal approach that combines OCT imaging technique and various methods of optical spectroscopy. This method also could be used for the improved lymphangiography of human *in vivo* skin in real time.

## 4 Conclusion

We presented the results of a comparative study of the OC effect by biocompatible mixtures of OCA and CPEs. Polyethylene glycol-400 as well as aqueous solutions of glucose and sucrose were used as OCA. Propylene glycol, oleic acid, and DMSO were used as CPEs. To enhance the clearing effect, physical permeation methods, such as dermabrasion and sonophoresis, were used. By analyzing the R ratio, which contains information about the average intensity and contrast of the images obtained by LC-OCT method, we determined the in-depth effectiveness of skin *in vivo* OC using various OCA. The results showed that the overall level of the R ratio in the 70- to 400-μm skin depth region, calculated as the AUC, showed the best increase (40%) after 10 min of US-assisted clearing using a mixture of polyethylene glycol-400/oleic acid/propylene glycol. The other eight OCAs also showed an increase in the overall R level with depth. The results of the experimental data fitting with the hypothesized biphasic exponential association model are in a good agreement with experimental results. Thus, the effectiveness of OC with biocompatible concentrations of OCA was proven through an increase in the overall intensity and contrast of the obtained LC-OCT images over the entire depth of the examined skin *in vivo*.

## Data Availability

The data that support the findings of this study are available from the authors on reasonable request.
